# *Picochlorum celeri* as a model system for robust outdoor algal growth in seawater

**DOI:** 10.1038/s41598-021-91106-5

**Published:** 2021-06-02

**Authors:** Anagha Krishnan, Maria Likhogrud, Melissa Cano, Scott Edmundson, Jenna B. Melanson, Michael Huesemann, John McGowen, Joseph C. Weissman, Matthew C. Posewitz

**Affiliations:** 1 Department of Chemistry, Colorado School of Mines, Golden, CO 80401 USA; 2 Marine and Coastal Research Laboratory, Pacific Northwest National Laboratory, Sequim, WA 98382 USA; 3 Arizona Center for Algae Technology and Innovation, Arizona State University, Mesa, AZ 85212 USA; 4 Corporate Strategic Research, ExxonMobil, Annandale, NJ 08801 USA

**Keywords:** Biotechnology, Environmental biotechnology

## Abstract

With fast growth rates, broad halotolerance and the ability to thrive at high temperatures, algae in the genus *Picochlorum* are emerging as promising biomass producers. Recently, we isolated a remarkably productive strain, *Picochlorum celeri*, that attains > 40 g m^−2^ day^−1^ productivities using simulated outdoor light. To test outdoor productivities, *Picochlorum celeri* was cultivated in 820 L raceway ponds at the Arizona Center for Algae Technology and Innovation. *Picochlorum celeri* demonstrated the highest outdoor biomass productivities reported to date at this testbed averaging ~ 31 g m^−2^ day^−1^ over four months with a monthly (August) high of ~ 36 g m^−2^ day^−1^. Several single day productivities were > 40 g m^−2^ day^−1^. Importantly for sustainability, *Picochlorum celeri* achieved these productivities in saline water ranging from seawater to 50 parts per thousand sea salts, without any biocides or pond crashes, for over 143 days. Lastly, we report robust genetic engineering tools for future strain improvements.

## Introduction

Marine microalgae are an attractive option for producing renewable biomass due to their ability to convert CO_2_ into complex products using simple nutrients, light and seawater^1–5^. *Picochlorum celeri* is a particularly fast-growing marine alga (photoautotrophic doubling time ~ 2 h under optimal growth conditions) that is halotolerant, thrives at high-light intensities [> 2000 μmol m^−2^ s^−1^ photosynthetically active radiation (PAR)] and is adapted to the elevated temperatures (~ 35–40 °C) often reached during domesticated pond growth^6^. This alga was isolated using high-irradiance and elevated-temperature selective pressures on photoautotrophic enrichments that were collected near Corpus Christi, Texas^6^. *Picochlorum celeri* has exceptional biomass productivities that exceed 40 g m^−2^ d^−1^ when cultured using diel conditions in laboratory photobioreactors running scripts that simulate outdoor light^6^.

Henley and co-workers were the first to describe the *Picochlorum* genus^7^, which has become the focus of several recent studies probing their high light, high salt, and high temperature tolerances, as well as their small, compact genome architectures^8–11^. The remarkable growth rates of *Picochlorum celeri* in seawater media is of particular interest for biofuels and other sustainable biotechnology applications^6^. Recently, a fully phased diploid genome sequence was assembled and reported^12^ for *Picochlorum celeri*, and a robust Cas9-ribonucleoprotein particle protocol was developed for high-efficiency genome editing in this alga^13^.

To determine whether the favorable growth rates and stress tolerances observed for *Picochlorum celeri* in the laboratory are maintained through scale-up from laboratory flasks to outdoor ponds, we used the U.S. Department of Energy (DOE) DISCOVR (Development of Integrated Screening, Cultivar Optimization, and Verification Research) program to attain growth metrics for *Picochlorum celeri* at larger scales. This pipeline uses indoor evaluation and testing at the Pacific Northwest National Laboratory (PNNL) to carefully map strain growth optima and to assess strain performance using outdoor growth simulators^14^. For promising strains, outdoor growth trials are performed at the Arizona Center for Algae Technology and Innovation (AzCATI) testbed. Using this DISCOVR pipeline, we report outdoor productivities for two consecutive summer outdoor growth campaigns (2019 and 2020). Additionally, we report the expansion of genetic manipulation tools for this alga.

## Results

### Temperature and dissolve salt profile

To determine whether the promising productivities previously reported in the laboratory^6^ could be scaled and realized outdoors, *Picochlorum celeri* was submitted to the DOE DISCOVR algal biotechnology program workflow^14^. Using the PNNL thermal gradient incubator and salt gradient incubator, temperature and salt optima of *Picochlorum celeri* were determined. First, the maximum specific growth rate was measured in dilute, exponential phase, shake-flask cultures grown in DISCOVR medium (35 PPT dissolved marine salts) and illuminated with white light at ~ 480 µmol m^−2^ s^−1^ PAR (Table 1). Each flask was exposed to a fixed temperature, ranging from 4 to 45 °C. Between 25 and 40 °C, *Picochlorum celeri* exhibited specific growth rates of > 4 day^−1^ (< 4 h doubling time) with a maximum of ~ 6.8 ± 0.2 day^−1^ (~ 2.5 h doubling time) at 35 °C. *Picochlorum celeri* growth rates were marginally higher at 40 °C than at 30 °C. Growth was significantly slower at temperature extremes below 13 °C and above 45 °C.

**Table 1:** Maximum specific growth rate of dilute *Picochlorum celeri* in DISCOVR medium, constant light and 35 PPT marine salt concentration as a function of temperature.

Temperature (°C)	Maximum specific growth rate (day^−1^)^a^	n^b^	t-test *p* value wrt 35.3 °C^c^
4.2	0.05 (0.04)	5	< 0.001
13.1	0.45 (0.15)	7	< 0.001
19.1	2.27 (0.22)	7	< 0.001
24.9	4.05 (0.17)	7	< 0.001
29.3	5.31 (0.14)	7	< 0.001
35.3	6.75 (0.20)	9	
40.5	6.08 (0.24)	9	0.05
45.5	1.18 (0.63)	6	< 0.001

^a^Standard error is given in brackets.

^b^n represents the number of replicates for each condition.

^c^Gives the t-test *p* value for each concentration with respect to growth rates at 35 °C.

As shown in Table 2, *Picochlorum celeri* was also grown in diel conditions in custom bioreactors using a solar simulation script approximating a clear day in mid-May (May 17) in Phoenix, AZ, as described previously^6^. Dense culture medium was specifically used to support the growth of high biomass *Picochlorum celeri* cultures^6^. Temperature scripts used were either (a) constant temperature of 33 °C, (b) ranging from a night low of 27 °C to a day high of 37 °C, or (c) ranging from a night low of 20 °C to day high of 35 °C, as indicated. Again, temperature was a significant factor influencing biomass productivities with the higher temperature regimes giving areal biomass productivities of ~ 45 g m^−2^ day^−1^; whereas the lower temperature script resulted in ~ 10% less biomass (t-test *p* value = 0.001) (Table 2).

**Table 2:** Diel biomass productivity of *Picochlorum celeri* at 35 PPT marine salt concentration in dense-culture medium as a function of temperature.

Temperature (°C)	Biomass productivity (g m^−2^ d^−1^)^a^	n^b^	t-test *p* value wrt 33 °C^c^
33	45.2 (0.8)	10	
27–37	45.2 (0.6)	7	1
20–35	39.3 (0.9)	6	< 0.001

^a^Standard error is given in brackets.

^b^n represents the number of replicates for each condition.

^c^Gives the t-test *p* value for each concentration with respect to growth rates at 33 °C.

Halotolerance was determined by quantifying maximum specific growth rates as a function of salt concentrations at 25 °C and constant light of ~ 480 µmol m^−2^ s^−1^ PAR (Table 3). Interestingly, *Picochlorum celeri* showed no statistical difference in growth rates at 50 PPT as compared to 35 PPT marine salts (µ (35 PPT) = 3.99 ± 0.41 day^−1^, µ (50 PPT) = 3.61 ± 0.31 day^−1^, t-test *p* value = 0.44) under the growth conditions used, and was able to tolerate salt concentrations up to 100 PPT (~ 3X seawater). These data demonstrate that *Picochlorum celeri* grows well at the elevated temperatures and salt concentrations frequently encountered in outdoor seawater ponds.

**Table 3:** Maximum specific growth rates of *Picochlorum celeri* at 25 °C as a function of marine salt concentration and grown in DISCOVR medium.

Salt concentration (PPT)	Maximum specific growth rate (day^−1^)^a^	n^b^	t-test *p* value wrt 35 PPT^c^
35	3.99 (0.41)	5	
50	3.61 (0.31)	6	0.45
75	2.86 (0.14)	6	0.03
100	2.03 (0.10)	6	0.001

^a^Standard error is given in the brackets.

^b^n represents the number of replicates for each condition.

^c^Gives the t-test *p* value for each concentration with respect to growth rates at 35 PPT.

*Picochlorum celeri* also showed robust growth across a range of marine-salt concentrations when tested at higher temperature (33 °C) in constant light (900 μmol m^−2^ s^−1^), and grown in the custom photobioreactors^6^ (Table 4). To measure specific growth rates, *Picochlorum celeri* was grown in dilute culture medium, specifically used for growing *Picochlorum celeri* at chlorophyll densities < 0.5 µg mL^−1^. Specific growth rates in marine salts ranging from 9 to 35 PPT were all at or above 7.4 day^−1^ and declined to 4.2 day^−1^ at 70 PPT (~ 2X seawater).

**Table 4:** Maximum specific growth rates of *Picochlorum celeri* in constant light (900 μmol m^−2^ s^−1^ PAR) at 33 °C in dilute-culture medium as a function of the concentration of Instant Ocean sea salts.

Salt concentration (PPT)	Maximum Specific Growth Rate (day^−1^)^a^	n^b^	t-test *p* value wrt 35 PPT^c^
9	7.4 (0.03)	4	0.01
18	7.9 (0.06)	4	0.002
35	7.6 (0.04)	11	
70	4.2 (0.5)	3	< 0.001

^a^Standard error is given in the brackets.

^b^n represents the number of replicates for each condition.

^c^gives the t-test *p* value for each concentration with respect to growth rates at 35 PPT.

### Outdoor cultivation

**Figure 1:**
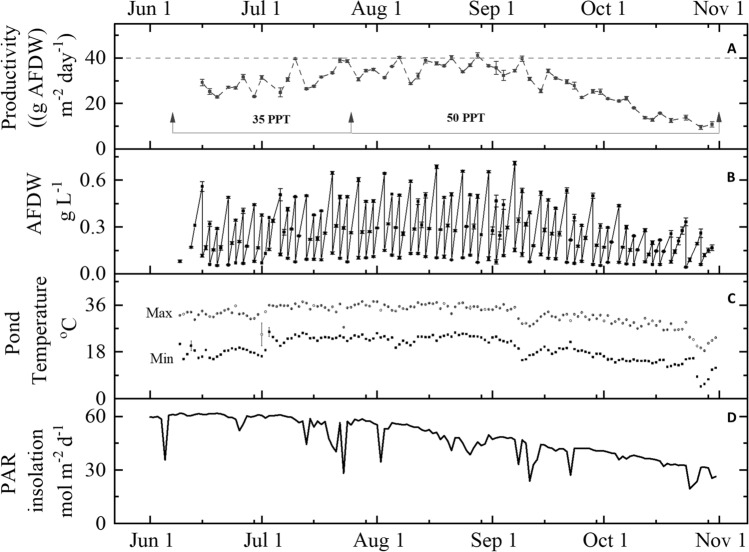
Outdoor pond productivity, temperature and light: (**A**) Harvest-to-harvest average areal biomass productivity, (**B**) volumetric AFDW, (**C**) daily minimum and maximum pond temperatures and (**D**) the daily PAR insolation for the 2020 outdoor field trial. Cultures were grown in nitrogen-replete medium in outdoor ponds located in Mesa, AZ. For areal productivity (**A**) and volumetric biomass concentration (**B**) and pond temperature (**C**), each datapoint represents the average of three ponds (n = 3) and standard error of mean. The period for which media salt concentrations are either 35 PPT or 50 PPT is shown by a horizontal line and arrowheads (in grey) in (**A**). Dotted line in (**A**) denotes 40 g m^−2^ day^−1^. PAR insolation (mol photons m^−2^ d^−1^) received by the ponds each day over the duration of the outdoor campaigns (**D**).

**Figure 2:**
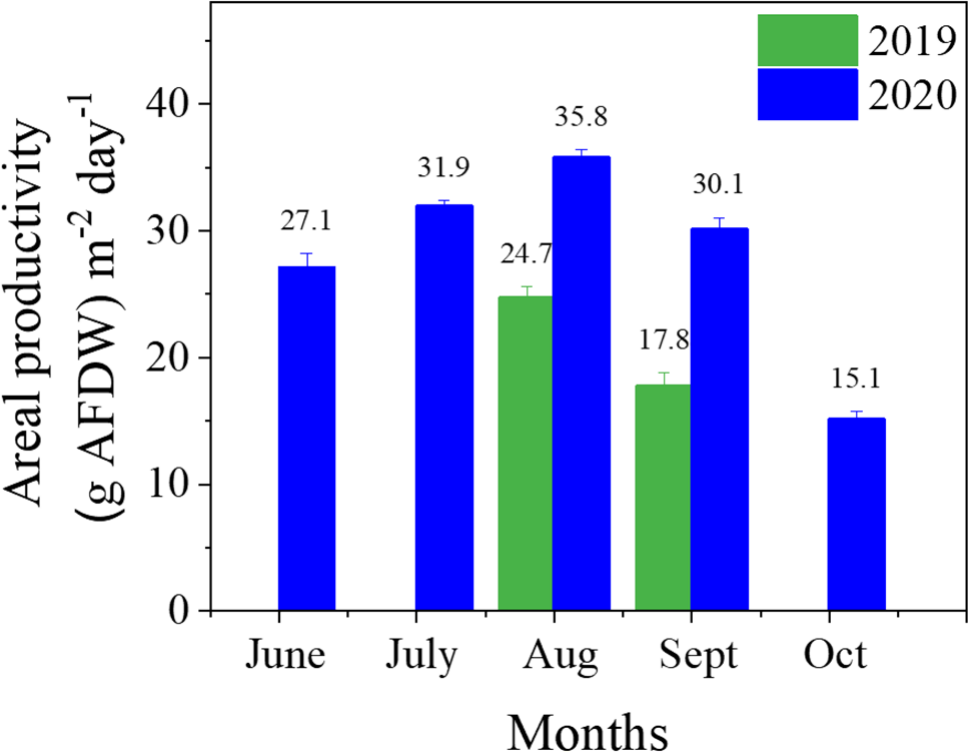
Monthly productivity: Average areal biomass productivity calculated on a monthly basis. For the three ponds, for each month, an average daily productivity was obtained. Data represent the averages and standard deviations of this monthly average from the three ponds.

In accordance with the DISCOVR pipeline, *Picochlorum celeri’s* growth metrics were initially assessed using the Laboratory Environmental Algae Pond Simulator (LEAPS) photobioreactor; an indoor photobioreactor system for evaluating promising new strains under climate simulated seasonal conditions^15^. Here, the cultures are exposed to a time series (called “scripts”) of incident solar radiation and pond temperatures generated by the ‘Biomass Assessment Tool (BAT)’ using 30-year meteorological data at a desired pond location on a predetermined day^16^. The LEAPS trials suggested that *Picochlorum celeri* had the potential of attaining outdoor productivities > 25 g m^−2^ day^−1^ at a pond located in Mesa, AZ. On the basis of these initial observations, *Picochlorum celeri* was advanced to outdoor pond testing at AzCATI in Mesa, AZ. AzCATI serves as a U.S. national algae testbed to facilitate algal biotechnology development and commercialization. Cultures were scaled indoors using bubble columns and 15 L flat panel reactors as described in McGowen et al.^17^ and finally inoculated into 820 L raceway ponds at a depth of 20 cm and a surface area of 4.2 m^2^. The ponds were run in triplicate, for 64 days in 2019 and 143 days in 2020 under semi-continuous growth conditions with samples harvested every 2 or 3 days along with dilution of the ponds at the time of each harvest. For the duration of the experiment, environmental parameters including PAR, pond and air temperature, relative humidity, dissolved oxygen and pH were monitored. Figure 1 shows data collected during the 2020 run including the calculated areal harvest-to-harvest productivity (gAFDW m^−2^ day^−1^) (Fig. 1A), volumetric biomass concentration (gAFDW L^−1^) (Fig. 1B), daily minimum and maximum pond temperature (°C) (Fig. 1C) and daily PAR insolation (mol photons m^−2^ d^−1^) (Fig. 1D). Monthly average productivity was calculated by dividing the total biomass harvested over the month by the area of the pond (4.2 m^2^) and the number of days the pond was run in each month. Monthly average productivity data collected for both 2019 and 2020 are given in Fig. 2.

As seen in Fig. 1, from June to the middle of September 2020, the pond temperatures remained in the 14–37 °C range, while October was significantly colder in the range of 4–32 °C (Fig. 1C). Daily PAR insolation was highest in June (~ 60 mol photons m^−2^ day^−1^) and then started to decrease through the middle of July, ultimately reaching as low as ~ 25 mol photons m^−2^ day^−1^ by the end of October (Fig. 1D). To test for the effect of salt concentration on productivity, media was changed from 35 to 50 PPT marine salts on August 9, 2019 and July 25, 2020 respectively.

In 2019, *Picochlorum celeri* was run in the AzCATI testbed for the months of August and September using the DISCOVR medium. Consistent with the LEAPS productivity data, *Picochlorum celeri* demonstrated a maximum average productivity of 21.2 ± 3.9 g m^−2^ day^−1^ in 2019 with August productivity (24.7 ± 0.9 g m^−2^ day^−1^) being higher than September (17.8 ± 1.0 g m^−2^ day^−1^) (Fig. 2). In 2020, *Picochlorum celeri* was run in the testbed for five months from June to October. During the 2020 run, medium composition was changed to a modified F/2 based medium as described in the Materials and Methods section. The major differences between the 2019 and 2020 media was a lower N:P ratio in 2020 (35.5:1 in 2019 vs 16:1 in 2020) with a higher overall amount of N (~ 1.5 X) and P (~ 3.5 X) as compared to the 2019 media. In 2020 reverse-osmosis water was used; whereas, in 2019 Mesa, AZ municipal water was used. In the 2020 run, *Picochlorum celeri* displayed average productivities of 27.1 ± 1.2 g m^−2^ day^−1^, 31.9 ± 0.5 g m^−2^ day^−1^, 35.8 ± 0.7 g m^−2^ day^−1^ and 30.1 ± 0.9 g m^−2^ day^−1^ in June, July, August and September respectively, with an overall run average for the four months of 31.2 ± 3.3 g m^−2^ day^−1^ (Fig. 2). Notably, even after the switch in marine-salt concentration from 35 to 50 PPT on July 25, 2020, *Picochlorum celeri* maintained high productivity and had multiple days with productivities surpassing 40 g m^−2^ day^−1^ (Fig. 1). While *Picochlorum celeri* was highly productive at the beginning of September, by the middle of September and through October, the productivity showed a steady decline in parallel with a decrease in both temperature and the available insolation (Fig. 1). Importantly, throughout the entire 2020 run, the ponds were naturally stable, exhibiting little or no contamination by protozoans or other algae (pond samples were routinely checked using microscopy), and did not require any biocide or herbicide treatments.

The above results clearly demonstrate the stability of *Picochlorum celeri* cultivation in small-scale outdoor ponds, especially during the summer months when temperatures and light levels are high. The demonstrated productivities from June–September 2020, are not only the highest reported for any alga grown at the AzCATI testbed site to date, but are among the highest outdoor productivities reported in the literature for any alga.

### Strain engineering

To support efforts probing the mechanistic underpinnings of the *Picochlorum celeri* productivities and stress tolerances, and to enable future engineering efforts, genetic manipulation tools were developed for the rapid generation of transformants expressing transgenes of interest.

### Antibiotic markers, transformation protocol

**Figure 3:**
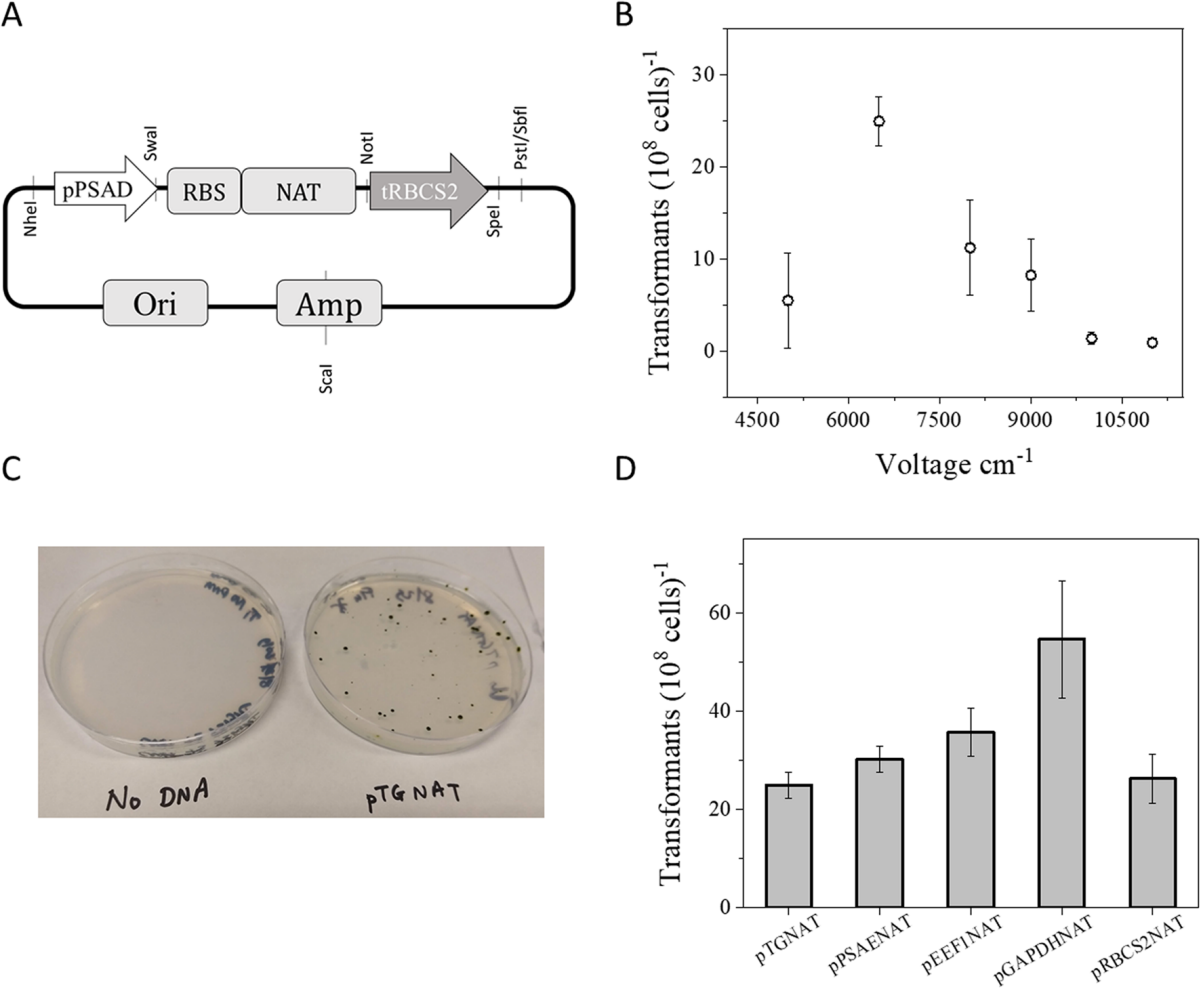
Optimization of electroporation methods: (**A**) Biobrick-style clonNAT resistance plasmid used for initial studies. Restriction sites are shown. RBS: ribosome binding site; Ori: Origin of replication; Amp: Ampicillin resistance marker, (**B**) Relative transformation efficiency under different field strengths (5000, 6500, 8000, 9000, 10,000, 11,000 Vcm^−1^), (**C**) clonNAT resistant colonies on selective agar plates with optimized parameters using *Sca*I linearized pTGNAT plasmid, (**D**) relative transformation efficiency of constructs expressing clonNAT resistance gene driven by various endogenous *Picochlorum celeri* promoters. Data represents the average and standard deviation for 3 technical replicates.

Several antibiotics were tested for their effects on exponentially growing *Picochlorum celeri* by spreading 10^8^ cells on antibiotic containing plates and checking for growth after 7 days. Nourseothricin (clonNAT) (> 50 mg L^−1^), G418 (> 400 mg L^−1^) and phleomycin (> 15 mg L^−1^) were found to be effective at suppressing growth under the conditions tested; zeocin (50 mg L^−1^), blasticidin (10–50 mg L^−1^) and hygromycin B (300 mg L^−1^) did not effectively kill all background cells (Figure S1). The clonNAT resistance marker gene (Genbank ARQ80408.1) was codon optimized for *Picochlorum celeri*^18^ and cloned between the *Picochlorum celeri* endogenous *PSAD* promoter and *RBCS2* terminator. The resulting plasmid, pTGNAT (Fig. 3A), was used as the base transformation construct to optimize electroporation parameters. Importantly, spreading colonies on top of agar plates resulted in a non-homogenous lawn of cells (Figure S1) with the appearance of false positive colonies upon prolonged incubation. Consequently, a soft-agar overlay (top-agar) method, which allows for a homogenous distribution of algal cells within a thin layer of agarose was used. The embedding technique allowed for (i) a gel-like moist environment for the cells to grow and (ii) more uniform exposure of the cells to the antibiotic selection pressure.

Electroporation based transformations of other *Picochlorum* strains were previously reported^9,10^; however, the protocols described did not yield positive transformants for *Picochlorum celeri*. As shown in Fig. 3B, successful transformation of *Picochlorum celeri* was attained using a pulse duration of 25 ms and a field strength range between 5000 Vcm^−1^ to 10,000 Vcm^−1^, (6500 Vcm^−1^ being optimal). Using a field strength of 6500 Vcm^−1^, 5 µg of linearized pTGNAT DNA and top-agar plating typically results in ~ 25 “pickable” colonies per 10^8^ cells after 14 days of incubation on selection plates (Fig. 3C). Colonies embedded lower in the soft agar layer take longer to grow, becoming visible after ~ 14 days and are not counted in our analysis.

### Gene promoters

Efficient expression of foreign genes is usually achieved by strong promoters^19^. In addition to *pPSAD*, two light-dependent (*pPSAE*, *pRBCS2*) and two light-independent (*pEEF1A* and *pGAPDH*) endogenous promoters were used to drive the expression of the clonNAT resistance marker gene and the resulting constructs were transformed into *Picochlorum celeri* (Fig. 3D, Table 5). Relative transformation efficiencies of the tested promoter constructs were similar to pTGNAT, with the exception of pGAPDHNAT, which gave almost twofold higher number of colonies per 10^8^ cells (55 ± 12). Random integration of the clonNAT marker into the nuclear genome was confirmed using genome walking PCR (data not shown)^20^.

**Table 5:** Plasmids used in this study.

Plasmid name	Promoter	Gene of interest	Terminator	Purpose
pAK1A				Biobrick-style scaffold vector using pUC19 backbone. Contains MCS for cloning in genetic elements (Figure S2A)
pAK1B	*PSAE*		*GAPDH*	Chassis plasmid with *PSAE* promoter and *GADPH* terminator built in pAK1A
pTGNAT	*PSAD*	clonNAT resistance	*RBCS2*	Encodes clonNAT resistance gene for selection of transformants (Fig. 3A)
pPSAENAT	*PSAE*	clonNAT resistance	*GAPDH*	Encodes clonNAT resistance gene for selection of transformants
pEEF1NAT	*EEF1A*	clonNAT resistance	*RBCS2*	Encodes clonNAT resistance gene for selection of transformants
pGAPDHNAT	*GAPDH*	clonNAT resistance	*RBCS2*	Encodes clonNAT resistance gene for selection of transformants
pRBCS2NAT	*RBCS2*	clonNAT resistance	*RBCS2*	Encodes clonNAT resistance gene for selection of transformants
pAK7	*CAMV35S*	*bfloGFP*	*CAMV35S*	Encodes *GFP* driven by *CAMV35S* promoter and terminator (Figure S2B)
pAK8	*PSAE*	*bfloGFP*	*GAPDH*	Encodes *bfloGFP* driven by *PSAE* promoter and GAPDH terminator
pAK9	*PSAE*	*bfloGFP*	*GAPDH*	Expressed *bfloGFP* with a chloroplast targeting signal
pAK10	*PSAE*	*bfloGFP*	*GAPDH*	Expresses *bfloGFP* with a periplasm targeting signal
pAK11d	*PSAD, PSAE*	*NAT, bfloGFP*	*RBCS2, GAPDH*	Encodes *bfloGFP* driven by *PSAE* promoter and *GAPDH* terminator inserted pTGNAT backbone (Figure S2C)
pAK12d	*PSAD, PSAE*	*NAT, bfloGFP*	*RBCS2, GAPDH*	Encodes chloroplast targeted *bfloGFP* driven by *PSAE* promoter and *GAPDH* terminator inserted pTGNAT backbone (Figure S2D)
pAK13d	*PSAD, PSAE*	*NAT, bfloGFP*	*RBCS2, GAPDH*	Encodes periplasm targeted *bfloGFP* driven by *PSAE* promoter and *GAPDH* terminator inserted pTGNAT backbone (Figure S2E)

### Reporter genes and localization

To test for functional heterologous protein production in *Picochlorum celeri*, the sequence coding for the green fluorescent protein (bfloGFP) from *Branchiostoma floridae*^21^ was inserted in between the *PSAE* promoter and *GAPDH* terminator in tandem with the clonNAT resistance marker gene driven by the *PSAD* promoter (pAK11d) (Figure S2, Table 5). The chloroplast transit sequence from the soluble starch synthase gave a relatively high chloroplast localization score when analyzed using TargetP (http://www.cbs.dtu.dk/services/TargetP/). Therefore, this leader sequence was chosen for the generation of a chloroplast-targeting vector that allows the fusion of the signal peptide to transgenes of interest (Figure S2D). The signal peptide corresponding to the secreted arylsulfatase *ARS1* gene in *Picochlorum celeri* was identified using TargetP and used for targeting GFP to the periplasmic membrane (Figure S2E). Arylsulfatase is a periplasmic protein that is released into the culture medium in a cell wall-less mutant of *Chlamydomonas reinhardtii*^22^.

**Figure 4:**
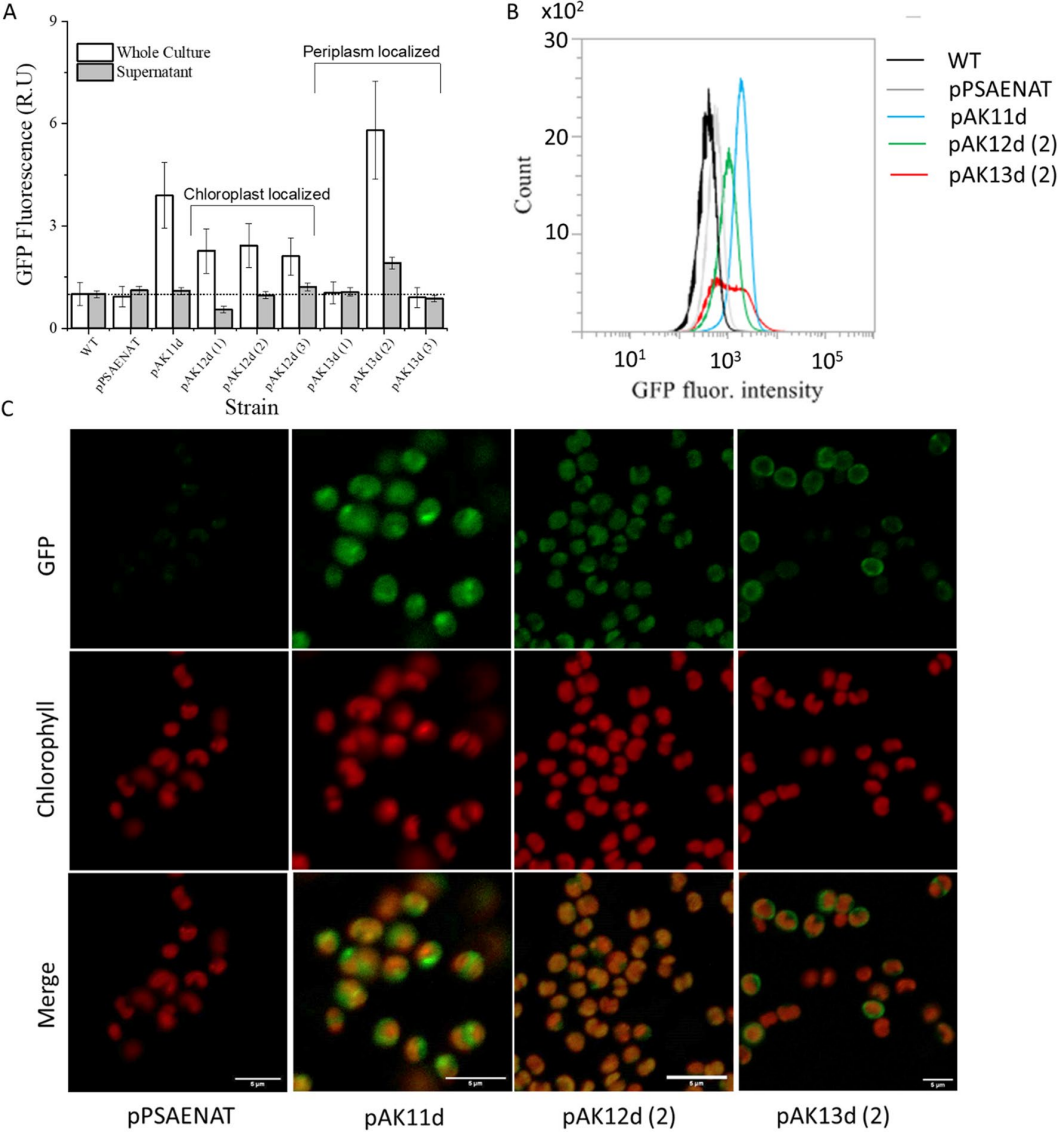
Phenotypic analysis of GFP expressing strains: (**A**) GFP fluorescence of whole culture and cell-free supernatant using a plate reader (excitation (485/20), emission (520/20)). Raw data was first normalized to OD_600_ for each culture as measured by the plate reader followed by a second normalization to the WT GFP signal. Data represent averages and standard deviations of three technical replicates. (**B**) Flow cytometry histograms for cell counts vs. GFP fluorescence intensity (BL1 channel, 530/30 nm) for the control strains (WT, pSAENAT) and the maximum GFP expressing transformant clones i.e. pAK11d, pAK12d (#2), pAK13d (#2) for the cultures used in (**A**). (**C**) Confocal microscopy images. 5 µm scale bar shown in the bottom panel.

Fusions of the starch synthase or *ARS1* transit peptides to the 5′ end of GFP gene, resulted in gene expression vectors enabling the targeting of GFP to either the chloroplast (pAK12d) or the periplasmic space (pAK13d), respectively. A single *Picochlorum celeri* isolate transformed with pAK11d, and three clones each of pAK12d or pAK13d were picked, PCR verified for both *GFP* and clonNAT resistance genes, and characterized for GFP fluorescence using a microplate reader, flow cytometer and confocal microscopy (Fig. 4). The Cauliflower Mosaic Virus 35S Promoter driving GFP expression in tandem with the clonNAT marker was also tested but failed to show any GFP fluorescence (data not shown). Untransformed *Picochlorum celeri* and a strain transformed with the pSAENAT plasmid were used as negative (non-GFP) controls.

Microtiter plate based analyses of 100 µL whole-cell culture aliquots of pAK11d (untargeted GFP) and pAK13d (#2) (periplasmic space targeted GFP) gave 3.9 (± 0.9) and 5.8 (± 1.4)-fold higher GFP fluorescence (Ex. 485/20 nm Em: 520/20 nm) compared to the WT (Fig. 4A). All three pAK12d clones gave similar twofold higher fluorescence relative to control lines. pAK13d (#2) displayed fluorescence in the cell-free supernatant not shown by any other transformant suggesting the presence of secreted GFP in the media. pAK13d (#1, #3) did not show any fluorescence even though a GFP band was detected via PCR suggesting potential positional effects or transgene silencing^19,23^. pAK11d, pAK12d (#2) and pAK13d (#2) show a clear separation in the GFP fluorescence peak as compared to the non-GFP strains when analyzed using flow cytometry (Fig. 4B).

Confocal microscopy was performed to localize GFP in the transformed lines (Fig. 4C). GFP fluorescence is depicted in green and chlorophyll autofluorescence from the chloroplast in red. Cells transformed with pAK11d showed intense GFP fluorescence especially outside of the chlorophyll fluorescing region validating successful GFP expression and protein presence in the cytosol. In pAK12d, the red and green signals overlapped, with minimal GFP fluorescence outside the red portion indicating localization to the chloroplast. In pAK13d, a more intense GFP fluorescence on the periphery of the cells suggests that the use of the ARS signal peptide successfully helps target GFP predominantly to the periplasmic space. Along with our previously established ability to edit the *Picochlorum celeri* genome using CAS9^13^, these results demonstrate our capacity to perform targeted expression of heterologous proteins in this alga.

## Discussion

Internationally, several efforts are focused on the use of microalgal biomass for biofuels, foods, feeds, and other biocommodities. Biomass productivity increases and process stability improvements will benefit most of these applications^24^. Strains chosen for large-scale outdoor growth must be robust, tolerating extreme environments and exhibiting high productivity and longevity. In this context, we previously established an enrichment strategy to select strains with high photosynthetic activities and specific growth rates when exposed to high light and high temperature in marine media; conditions anticipated during outdoor mass cultivation. A new species of the genus *Picochlorum, Picochlorum celeri*, with very short doubling times, the capacity to grow under illumination of > 2000 µmol m^−2^ s^−1^ and diel productivities of ~ 40 g m^−2^ d^−1^ in indoor light simulated photobioreactors, emerged from these selections. The data presented here validates our enrichment strategy with *Picochlorum celeri* demonstrating a productivity of 35.8 g m^−2^ day^−1^ in August 2020 in the outdoor AzCATI testbed. Additionally, the ponds were stable demonstrating an average sustained productivity of 31.2 g m^−2^ day^−1^ for 111 consecutive days (June–September, 2020). Remarkably, throughout the outdoor campaign *Picochlorum celeri* attained peak productivities above 40 g m^−2^ day^−1^ on the best biomass production days (Fig. 1); in line with the previous laboratory bioreactor datasets^6^. Not only are these the highest outdoor metrics for any microalga cultivated at the AzCATI testbed, but also, to the best of our knowledge, the only strain reported to date to have sustained high productivity in marine salts at the level of 50 PPT (Table 6)^5,25–28^.

**Table 6:** Outdoor productivities of various high-performance algae for at least 30 days.

Alga	Phylum	g m^−2^ d^−1^ (Average)	Days in culture	Marine-salt concentration	Location	Reference	Notes
*Tetraselmis* sp.	Chlorophyta	37.5	78	15–35 PPT	Hawaii, USA	^34^	Not reproduced in subsequent work^35^
*Picochlorum celeri* (August 2020 only)	Chlorophyta	35.8	31	35–50 PPT	Mesa, AZ, USA	This work	Putatively best month due to higher temperatures
*Picochlorum celeri* (June – September 2020)		31	111	35–50 PPT	Mesa, AZ, USA	This work	Increased N and P; RO water relative to 2019 run
*Picochlorum celeri* (August – September 2019)		21	54	35–50 PPT	Mesa, AZ, USA	This work	
*Scenedesmus UTEX393*	Chlorophyta	27.1	85	5 PPT	Mesa, AZ, USA	^24^	Used fungicide to sustain growth performance at higher temperature
*Cyclotella* sp.	Ochrophyta	30	400	7–15 PPT	Hawaii, USA	^36^	Used foil arrays to allow systematic mixing
*Nannochloropsis* sp.	Ochrophyta	25	60		Israel	^44^	
*Chaetoceros muelleri*	Ochrophyta	32	125	35 PPT	California, USA	^45^	
*Cyclotella* sp.	Ochrophyta	32	43	8–20 total dissolved solids (TDS)	California, USA	^45^	Used hard water for growth
*Spirulina platensis*	Cyanobacteria	25–27 (DW)	360		Israel	^46^	Reported unashed dry weight

Productivity of any alga in open ponds is highly dependent on the seasonal and diurnal variations in temperature and insolation. Higher productivities were observed in the warmer months of July and August 2020 as opposed to the cooler month of June, even though the later exhibited the highest daily insolation (Fig. 1). While June had an average temperature maxima of ~ 32.5 °C, the minima was ~ 18 °C, dropping below the optimal temperature for *Picochlorum celeri* growth (Table 1). Besides affecting important aspects of growth e.g., respiration, photosynthetic rates etc., the lower suboptimal morning temperatures can also lead to increased photoinhibition in outdoor microalgal cultures^29^. At productivities of ~ 31 g m^−2^ day^−1^, *Picochlorum celeri* metrics are comparable if not higher than those of highly productive agricultural crops. For context, during their peak growing season, sugarcane and corn have been reported to produce 17–20 g m^−2^ day^−1^ ^30,31^ and 32 g m^−2^ day^−1^ ^32^ respectively of total dry biomass. *Picochlorum celeri* had multiple high productivity days of over 40 g m^−2^ day^−1^ during the 2020 outdoor growth campaign despite relatively little outdoor culturing experience and operational optimization being developed to date for this alga. Importantly, *Picochlorum celeri* has the ability to grow on non-arable land using seawater and did not require expensive and regulated crop management interventions such as herbicide and pesticide applications to attain the high yields reported, which is promising from a sustainability perspective. Lastly, along with good pond management strategies, careful siting of algal ponds at locations with favorable growth temperatures and sunlight, may enable high productivities to be attained throughout most of the calendar year (not limited to a single growing season) for optimized annual biomass yields.

*Picochlorum celeri* is a recent isolate that we are exploring for biofuel and bioproduct applications, and we have only had two limited outdoor growth campaigns to date. Both of these efforts showed the potential of this alga as a top-tier biomass production strain, but the 2020 results were more impressive relative to the 2019 runs. Several variables existed between the 2019 and 2020 summer growth campaigns and the precise reasons for different productivities are not yet entirely clear. We hypothesize that nitrogen in 2019 was limiting and insufficient to match the *Picochlorum celeri* demands under high-productivity conditions. As described above, nitrogen levels were increased 1.5X in the 2020 runs. Two additional variables between the 2019 and 2020 runs are noteworthy. First, the water resource changed from municipal water in 2019 to RO water in 2020. The 2019 cultures were clumped (potentially due to water hardness), whereas the 2020 cultures were not. Second, the phosphorous levels were increased 3.5X in 2020 and the N:P ratio changed from 35.5:1 (2019) to 16:1 (2020). Clearly, multiple variables (including distinct climatic conditions) existed between the 2019 and 2020 runs. Nevertheless, both campaigns showed promising productivities and stabilities. Additional outdoor runs and user experience will allow us to systematically isolate and test a multitude of variables, and these efforts are likely to lead to further increases in biomass productivity.

Among the most promising aspects of *Picochlorum celeri* are its tolerance to high temperature (up to 40.5 °C) and dissolved marine salts (100 PPT salt) (Tables 1, 2, 3, 4). Large open ponds, especially in warmer climates, are prone to significant evaporative losses leading to an increase in salt concentrations. Sustainability is reduced if salt concentrations are brought back to optimal levels by adding fresh water. Using saline water to make up for evaporation leads to increased salt levels in the ponds, which is controlled by disposing a portion of the pond volume (blowdown)^33^. *Picochlorum celeri’s* tolerance to high-salt concentrations minimizes the requirement for make-up water additions and hence overall biomass production costs. Additionally, salt levels can be manipulated for the management of contamination in open ponds rather than toxic biocides.

Shown in Table 6 are some of the highest productivity runs ever reported for outdoor algal growth campaigns. Unfortunately, many of these studies were done decades ago using strains (and facilities) that are no longer available. Several of these high performance runs were from a site in Hawaii^34–36^ that had particularly favorable temperatures, solar conditions and raceway designs for outdoor algal growth. The growth results for *Picochlorum celeri* are likely to improve beyond the yields reported here if future growth campaigns take full advantage of similar growth assets. We are only beginning outdoor growth campaigns with *Picocholorum celeri* and user experience combined with media, harvesting and siting improvements are likely to further increase current biomass productivities.

The theoretical limit for photosynthetic solar-to-glucose conversion efficiency has been calculated to be ~ 12% for total solar radiation and ~ 24% of PAR^37,38^. Assuming an average energy density of 23 kJ g^−1^ of algal biomass^39^ and 217 kJ mol^−1^ photons (PAR), the photosynthetic efficiency of *Picochlorum celeri* for the month of August 2020, was 7.7% equivalent to ~ 3.8% total solar radiation. Development of synthetic biology tools for *Picochlorum celeri* should allow progress towards greater photosynthetic efficiencies in supersaturating light, as well as to engineer other desirable traits (e.g. synthesis of high value coproducts). In this research, a library of genetic parts and methods facilitating rapid generation of the desired transformants in *Picochlorum celeri* was developed (Figs. 3, 4). Given the fast growth rates, transformants expressing heterologous proteins can be generated in less then two weeks. In this alga, nuclear transformation primarily occurs via random insertion of the DNA through non-homologous end joining. We also provide a range of usable promoters and a set of validated vectors for targeting transgene products to specific subcellular locations using transit peptides inherent to *Picochlorum celeri*. Using a periplasm signal peptide, we were able to target GFP for secretion. Combined with our previously reported CRISPR-Cas9 genome editing method^13^, the techniques reported here for the successful expression of heterologous proteins provide additional genetic tools for probing and manipulating the metabolism of *Picochlorum celeri*.

Clearly, with broad tolerances to high light, halotolerance, thermotolerance, high productivities and stabilities, *Picochlorum celeri* is capable of producing among the most promising biomass yields reported to date using non-potable water resources. Through two outdoor field trials, the marine alga *Picochlorum celeri* showed robust stability in outdoor ponds (~ 143 days) along with exceptional monthly (35.8 g m^−2^ day^−1^) and daily (> 40 g m^−2^ day^−1^) peak productivities.

## Materials and methods

### Algal culturing (DISCOVR pipeline)

For all the experiments conducted in the DISCOVR pipeline, including salt and temperature tolerance studies, LEAPS analysis and 2019 outdoor cultivation, DISCOVR media were used. The media were composed of 1.51 mM (NH_4_)_2_SO_4_, 0.09 mM (NH_4_)_2_HPO_4_, 3.57 mM NaHCO_3_, 37 pM cyanocobalamin, 11.7 µM FeCl_3_.6H_2_O, 11.7 µM Na_2_EDTA.2H_2_O, 39.3 nM CuSO_4_.5H_2_O, 26 nM Na_2_MoO_4_.2H_2_O, 76 nM ZnSO_4_.7H_2_O, 42 nM CoCl_2_.6H_2_O, 910 nM MnCl_2_.4H_2_O and either 39.2, 56, 84, or 112 g of Crystal Marine mix (Bioassay Laboratory Formula, Marine Enterprises International, LLC) added to 1 L medium to adjust the salt concentration to 35, 50, 75, or 100 PPT, respectively. For 2020 outdoor AzCATI campaigns, *Picochlorum celeri* was cultured in a modified F/2 media containing: 5.0 mM NH_4_HCO_3_, 0.3125 mM NaH_2_PO_4_, 11.7 µM FeCl_3_.6H_2_O, 11.7 µM Na_2_EDTA.2H_2_O, 39.3 nM CuSO_4_.5H_2_O, 26 nM Na_2_MoO_4_.2H_2_O, 76.5 nM ZnSO_4_.7H_2_O, 42 nM CoCl_2_.6H_2_O, 910 nM MnCl_2_.4H_2_O. 38.5 g of Instant Ocean Sea Salt was added to 1L of medium to adjust the salt concentration to 35 PPT, or 55 g of Instant Ocean Sea Salt was added to 1L of medium to adjust the salt concentration to 50 PPT. The water resource used for outdoor cultivation in 2019 was Mesa, AZ municipal water. The 2020 outdoor cultivation trial utilized reverse osmosis water.

Where indicated (Tables 2, 4), custom bioreactor growth experiments were done using either dilute culture medium supporting growth of cultures at a target chlorophyll density of < 0.5 μg mL^−1^, or in dense culture marine medium as described previously in Weissman et al.^6^.

### Measurement of specific growth rate as a function of salt concentration and temperature

For determining temperature dependent growth productivities, *Picochlorum celeri* cultures were grown in 125 mL Erlenmeyer flasks in DISCOVR medium and cultivated within PNNL’s Thermal Gradient Incubator. The temperatures tested range from 4 to 46 °C, with 6 intermediate points. For salt tolerance, DISCOVR medium was modified by adding artificial sea salts to generate a dissolved salt gradient of 35 PPT, 50 PPT, 75 PPT, and 100 PPT. Cultures were grown within PNNL’s Salinity Gradient Incubator at 25.5 ± 0.3 °C. For both incubators, illumination was provided by neutral white (4000 K) LED panels at ~ 450 µmol m^−2^ s^−1^, set to a 12:12 light:dark photoperiod. The actual light intensity within each flask at the surface of the culture medium was 480 ± 7 µmol m^−2^ s^−1^. Cultures were covered with a foam stopper and sparged with sterile-filtered, CO_2_-enriched (0.5% v/v) humidified air.

Precultures were acclimated to the desired salt concentration or temperature pressures for 48 h prior to the assay. Growth curves were initiated by diluting the culture below an optical density of 0.1 (OD_750_). Measurements were recorded over the course of the light period in optically thin cultures, with at least three time points per photoperiod. Maximum specific growth rates (µ_max_) were calculated by taking the slope of the natural log-transformed OD_750_ along a minimum of 3 time points. Slopes which had fit values (r^2^) less than 0.95 were disregarded. Growth curves were repeated 3–7 times for each cultivation condition.

Diel productivity data as a function of temperature (Table 2) was obtained from dense cultures diluted automatically by 60% each morning prior to light coming on. The reactors were illuminated using a solar day modeled after May 17 in Phoenix, AZ with a light duration of ~ 13.5 h from sunrise to sunset. Temperature profiles were programmed as summarized in the table. 0.75% CO_2_ in air was bubbled continuously into the 400 mL culture volume at 0.75 to 1 v/v/min, keeping the pH between 7.0 and 7.5. The culture vessels were Pyrex square bottles of 500 mL total volume.

For data presented in Table 4, cultures were diluted daily to keep total chlorophyll below 0.5 µg mL^−1^ as previously described^6^. Instant Ocean salt mix was added to attain the desired marine-salt concentrations. Specific growth rates given are based on CHN analyses^6^.

### Outdoor Arizona testbed pond cultivation: large scale

Three identical raceway ponds were utilized for the summer cultivation of *Picochlorum celeri*. Each pond surface area was ~ 4.2 m^2^ with a nominal volume of 820 L at a depth of 20 cm (Commercial Algae Professionals, http://www.commercialalgae.com). The ponds were equipped with a YSI 5200A-DC (YSI Inc., Yellow Springs, OH, USA) water quality monitoring system simultaneously measuring pH, pond water temperature (°C), dissolved oxygen saturation (%), and dissolved salt concentrations (g L^−1^). Ponds were also equipped with a stainless-steel paddle wheel and a CO_2_ sparge line connected to a Sweetwater ceramic diffuser (Model# DYPFP4, Pentair Aquatic Ecosystems) for pH control linked to the YSI online pH probe. Each pond was monitored using a combination of the online equipment described above and daily manual grab samples for tracking biomass productivity (optical density, dry weight (DW) and ash-free dry weight (AFDW), nutrients, and optical microscopy as described in^17^. Weather data is recorded on site at the AzCATI testbed using a HOBO RX3000 Weather Station, equipped with air temperature, relative humidity, PAR, global light energy, rain and wind speed and direction sensors (Onset Computer Corporation, USA). PAR insolation (mol photons m^−2^ d^−1^) received by the ponds each day over the duration of the outdoor campaigns was calculated by integrating the measured PAR over the entire day.

Inoculum for the outdoor cultivation was produced in the same media as described for outdoor ponds. Once generated, the inoculum was transferred to outdoor ponds as described in McGowen et al.^17^. Ponds were operated with 24-h paddlewheel mixing at 7.2 rpm creating an average flow rate of 9.3 cm s^−1^ with on-demand CO_2_ sparging at 5 L min^−1^ based on a pH set point of 7.0. Although carbon uptake efficiency is an important metric in terms of algal cultivation, the cultivation is setup to ensure CO_2_ was not a limiting reagent and was supplied in excess. While the flow rate of CO_2_ to the system was controlled through a pH setpoint, actual volume delivered was not quantified. The ponds were operated in a semi-continuous, drain and fill, mode of operation whereby three (3) times a week a portion of the culture volume was removed on Monday, Wednesday and Friday mornings. Fresh media was added back to the remaining culture, the culture was allowed to mix, and the cultivation was allowed to continue. For the 2019 cultivation trial, the average volume percent removed was ~ 76% for August and September, and for the 2020 cultivation trial, the average volume percent removed was 80% for June through September, dropping to 75% for October.

### Algal culture conditions (strain engineering)

For strain engineering, a medium based on the NCMA (National Center for Marine Algae and Microbiota, USA) F/2 formulation^40,41^ was used. Specifically, the medium (henceforth called (Quarter sea-salt Algae Transformation Medium (QATM)) contained: ¼ volume/volume filtered seawater (NCMA), 4.4 mM urea, 434.4 µM NaH_2_PO_4_.H_2_O, 23.4 µM FeCl_3_.6H_2_O, 23.4 µM Na_2_EDTA.2H_2_O, 78.6 nM CuSO_4_.5H_2_O, 52 nM Na_2_MoO_4_.2H_2_O, 153 nM ZnSO_4_.7H_2_O, 84 nM CoCl_2_.6H_2_O, 182 nM MnCl_2_.4H_2_O, 148 µM thiamine HCl, 10.25 nM biotin, 184 nM cyanocobalamin and 5 mM MOPS (pH 7.6). 20 mM MOPS was used for solid (1.5% agar) and soft agar (0.4% low melt agarose) media. Liquid cultures were grown in a 1% CO_2_ chamber at 33 °C using a constant light (400–600 µmols m^−2^ s^−1^) regime and orbital shaking (110 rpm). Antibiotics G418 (500 mg L^−1^), nourseothricin (clonNAT) (75 mg L^−1^) and phleomycin (20 mg L^−1^) were supplemented in the solid medium wherever required.

### Vector design, codon usage and ribosome binding site

Plasmids and oligonucleotides used in this study are listed in Table 5 and Table S1. Using a pUC19 backbone, a BioBrick-style scaffold plasmid, pAK1A, was developed to contain a multiple cloning site (MCS) “*Nhe*I-*Swa*I-*Not*I-*Spe*I-*Pst*I” for easy insertion and switching of genetic elements (Figure S2A). The plasmids described below were constructed using this backbone vector. Using the *Picochlorum celeri* genome sequence^12^, a codon usage table^42^ (Table S2) and a putative ribosome binding site (RBS) (*Geneious Prime*, consensus sequence finder tool) were established. Codon optimized gene sequences for clonNAT resistance, bfloGFP, Cauliflower mosaic virus 35S (CAMV35S) promoter and terminator were synthesized by ATUM DNA2.0, Menlo Park, CA, USA (Table 5). Endogenous promoter and terminator sequences were PCR amplified from *Picochlorum celeri* genomic DNA using corresponding primers with restriction overhangs. The plasmid pAK1B, derived from pAK1A but containing the *PSAE* promoter in tandem with *GAPDH* terminator was constructed using Gibson cloning^43^ and was used for easy insertion of the gene of interest between the PSAE promoter and the GAPDH terminator using the *Swa*I/*Not*I sites. GFP was excised from pAK7d and inserted in pAK1B to generate pAK8d. The entire *PSAE* promoter-*bfloGFP*-*GAPDH* terminator was excised (*Nhe*/*Pst*I) and moved into pTGNAT (*Spe*I/*Pst*I digest) to generate pAK11d (Figure S2C) using the biobrick strategy. pAK12d and pAK13d (Figure S2D&E) were similarly constructed. All plasmids were sequence verified.

### Cloning and DNA preparation

Lysogeny Broth (LB) containing ampicillin (100 mg L^−1^) and *Escherichia coli* strains, NEB DH10B and C2925 were used for routine cloning purposes. Plasmid DNA was extracted from *E. coli* using ZymoPURE II Plasmid Midiprep (Zymo Research), linearized using *Sca*I, *Ecor*V or *Pst*I and purified using the QIAquick PCR Purification Kit (Qiagen). The purified fragment was dialyzed to remove any residual salts and evaporated to dryness using a centrifugal vacuum concentrator. The pellet was resuspended in water to a final DNA concentration of 1 µg µL^−1^.

### Electroporation

Early exponential phase cultures (OD_750_ ~ 0.3–0.5) in QATM medium were collected by centrifugation at 4000 rpm for 10 min. The cell pellet was washed 4 times with sterile 375 mM sorbitol (8000 g × 2 min). Cells were then resuspended in 375 mM sorbitol to a final concentration of 5 × 10^6^ cells µL^−1^. An aliquot of 100 µL cell suspension was mixed with 5 µg linearized plasmid (1 µg µL^−1^) and kept on ice for 3 min. Using a 0.2 cm prechilled electroporation cuvette (Bulldog Bio, Inc.), the DNA/cell mix was pulsed once in a Gene Pulser Xcell (Biorad) using the “Time Constant” protocol, 25 ms time constant at 1300 V (6500 Vcm^−1^). Electroporated cells were resuspended in 1 mL QATM medium and incubated at 33 °C in 1% CO_2_ atmosphere, and ~ 25 µmols m^−2^ s^−1^ light intensity. After a 6 h recovery, 240 µL of the cells (~ 1.09 × 10^8^ cells), was mixed with 3 mL QATM soft agar containing 75 mg L^−1^ clonNAT and overlaid onto QATM plates containing 75 mg L^−1^ clonNAT. Plates were incubated at 50 µmol m^2^ s^−1^ PAR, 1% CO_2_ at room temperature for 6 days and then moved to 200 µmol m^−2^ s^−1^ PAR, 1% CO_2_, 33 °C for 8 more days. Individual colonies were then picked and patched onto fresh selective plates.

### Transformant screening and Colony PCR

A loopful of cells from the patched plates were resuspended in 50 µL deionized water. Garnet beads (1/100th of total volume) were added to the cell suspension and bead beating was performed for 50 s at 4800 strokes/min. Lysate was centrifuged at 13,000 rpm and 1 µL of the supernatant was used for PCR verification of the gene of interest (reaction volume, 20 µL). Primers for *PSAD* gene fragment were used as an internal PCR control when needed.

### Relative GFP quantification: plate assay and flow cytometry

Transformants were picked from the selective plate and inoculated into liquid QATM medium and grown for 2 days. 100 µL aliquots of both the whole culture and the cell-free supernatant were separately loaded onto Corning black sided clear bottom 96-well microplates. Optical density (OD_600_), GFP fluorescence (Ex: 485/20 nm, Em: 520/20) and chlorophyll fluorescence (Ex: 360/40 nm, Em: 680/30 nm) of each well was determined using Synergy Multi-Mode Reader (BioTek Instruments). Attune NxT acoustic focusing flow cytometer (ThermoFisher Scientific) was used to measure the relative per cell GFP (530/30 nm) and chlorophyll (695/40 nm) fluorescence. For both microplate assays and flow cytometry, chlorophyll fluorescence was measured simultaneously to control for potential differences caused by cell physiology/autofluorescence.

### Confocal imaging

1 mL of 2-day old cultures were pelleted and resuspended in 100 µL QATM medium. 5 µL of the culture was mixed with 5 µL of QATM media containing 0.4% agarose, placed on a slide and covered with a coverslip. Immobilized cells were imaged at the University of Colorado Anschutz Medical Campus Advance Light Microscopy Core using an adapted laser scanning inverted confocal microscope (Zeiss LSM 780; Carl Zeiss AG) with a ×40X/1.2NA water-immersion objective.

### Data analysis

Both standard deviation and standard error are used as indicated. Standard deviation is used to provide an estimate of the uncertainty of the sample data. Standard error is given when statistical inferences are calculated using *t*-tests.

## Supplementary Information

Supplementary Information.
