# Supplementary material for: Picochlorum celeri as a model system for robust outdoor algal growth in seawater
Source: Sci Rep. 2021 Jun 2;11:11649. doi: 10.1038/s41598-021-91106-5 (PMC8172913; doi:10.1038/s41598-021-91106-5)
Supplement: Supplementary file 1 — Supplementary Information. [file 41598_2021_91106_MOESM1_ESM.pdf]

Supplementary Figures

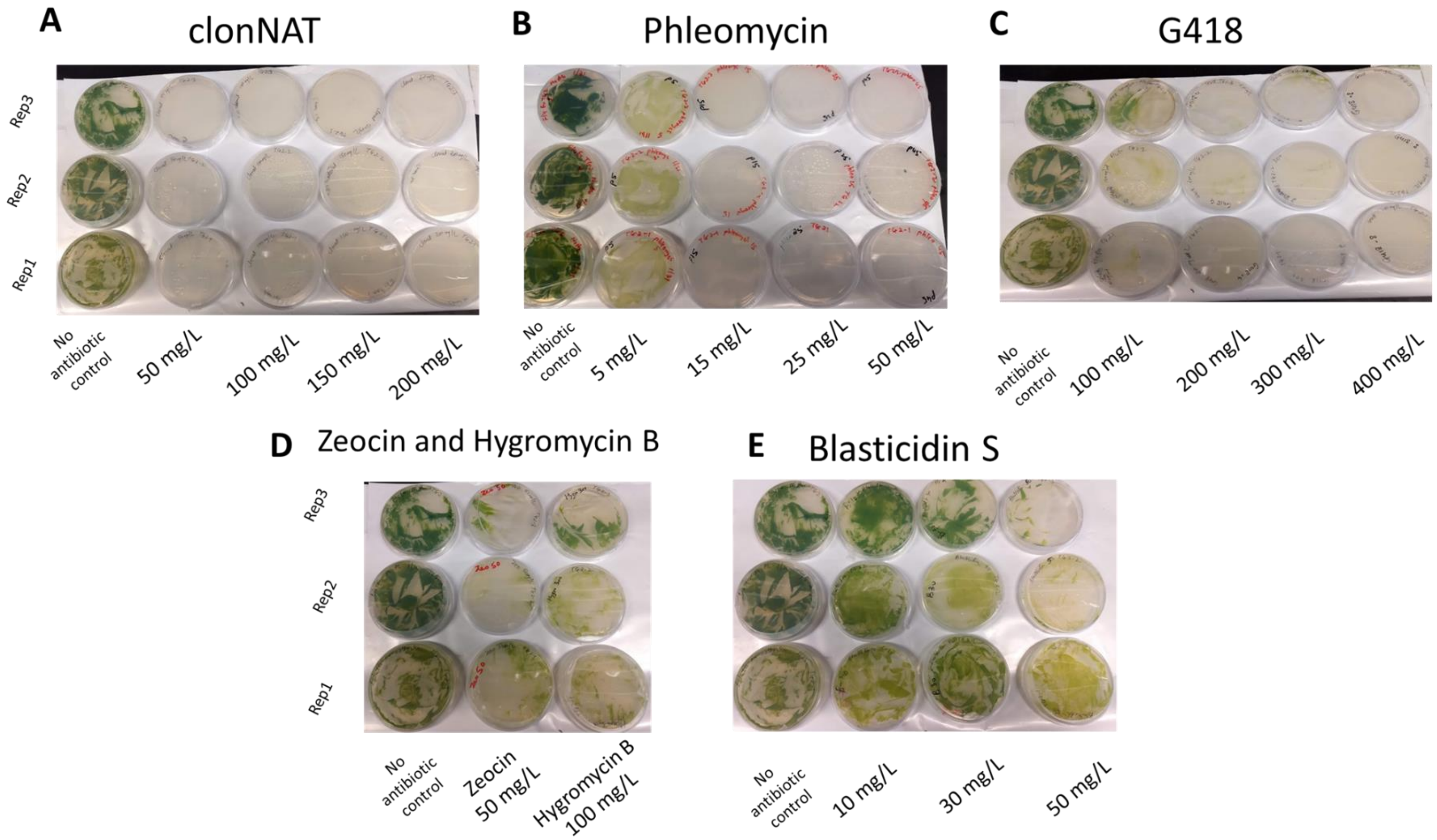

Fig. S1: Antibiotic kill curves: Growth of *P. celeri* after 7 days on QATM, 1.5% agar plates supplemented with antibiotics at varying concentrations. A) clonNAT (50-200 mgL^{-1}) B) Phleomycin (5-50 mgL^{-1}) C) G418 (100-400 mgL^{-1}) D) Zeocin (50 mgL^{-1}) and Hygromycin B (100 mgL^{-1}) and E) Blasticidin (10-50 mgL^{-1}). Kill curves were performed in triplicates. Antibiotic concentrations used are given at the bottom of each panel and replicate plate numbers are given on the left side of panel A and D.

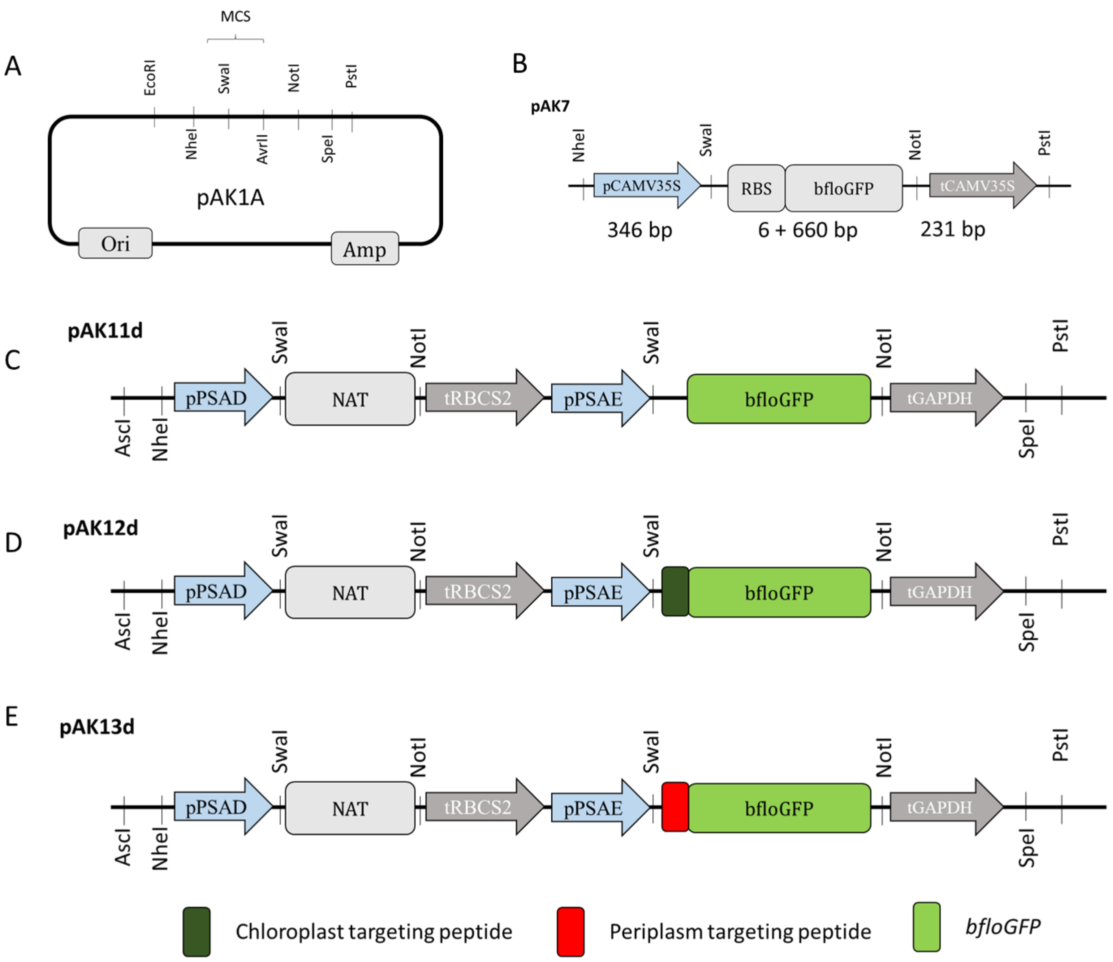

Chloroplast targeting peptide: MLAAQNTTKTFAFGTQNAIGRTSNYFGTSKVLKNASRVTRSASQRKSTVSVSAAV

Periplasm targeting peptide: MWHIYSSVALVLLLNTLGCAQATRE

Fig. S2: Plasmid constructs: Schematic representation of the plasmids constructed (linearized for the sake of representation) and targeting signals used in this study.

Supplementary Tables

Table S1: Primers used in this study for plasmid construction and insert verification

Primer name	Primer sequence: 5' --- 3'	Binding site	Purpose
AK_Fw	CCTCTGACTTGAGCGTCG	Binds in the pBR322 origin of replication area	Verification of gene insertion in pAK1A based plasmids
AK_Rev	GGTTATTGTCTCATGAGCGG	Binds in the Ampicillin resistance marker	Verification of gene insertion in pAK1A based plasmids
pAK1a_5	AAATCCTAGGGCGGCCGCACTAGTTGGGAGCCTGCAG	Partially binds pUC19 with restriction site overhands	Gibson primer for creation of pAK1A MCS
pAK1a_3	GCGGCCGCCCTAGGATTTAAATGCTAGCCTCTCCGAATTC	Partially binds pUC19 with restriction site overhands	Gibson primer for creation of pAK1A MCS
picoGAPDHpm5	AGATAGCTAGCCAGCCGTCAACGAGTCACG	Binds to the 5' end of the putative *GAPDH* promoter	For *GAPDH* promoter amplification
picoGAPDHpm3	ATACTATTTAAATAGACTTGATTGAGGTAGCA	Binds to the 3' end of the putative *GAPDH* promoter	For *GAPDH* promoter amplification
picoRBCS2pm5	AGATAGCTAGCCTCTGTTCTAAAGGTTCTG	Binds to the 5' end of the putative *RBCS2* promoter	For *RBCS2* promoter amplification
picoRBCS2pm3	ATACTATTTAAATTTGTTTGTAGTAAGTTTTCAA	Binds to the 3' end of the putative *RBCS2* promoter	For *RBCS2* promoter amplification
pEEF1A5	AGATAGCTAGCAATTCCAATTCCCGAATTAGTTT	Binds to the 5' end of putative *EEF1A* promoter	For *EEF1A* promoter amplification
pEEF1A3	ATACTATTTAAATAGAGATAGTTAACAACACTGAG	Binds to the 3' end of putative *EEF1A* promoter	For *EEF1A* promoter amplification

pGBtGAPDH5	GAACATTTAAATGCGGCCGCTGTC AAAGTAAGCAGTCAGC	Binds to the 5' end of the putative GAPDH terminator with overhangs for *pPSAE*	Gibson primer for constructing pAK1B
pGBtGAPDH3	CCTGCAGGCTCCCAACTAGTGACC TACTATCCAATTGTTG	Binds to the 3' end of putative *GAPDH* terminator with overhangs for pAK1A	Gibson primer for constructing pAK1B
pGBpPSAE5	CCGAATTCGGAGAGGCTAGCTGCA TTGCAACCAGGGTCAA	Binds to the 5' end of *PSAE* promoter with 5' overhangs for pAK1A	Gibson primer for constructing pAK1B
pGBpPSAE3	GACAGCGGCCGCATTTAAATGTTC AAGGTATTTATATGAA	Binds to the 3' end of PSAE promoter with overhangs for *tGAPDH*	Gibson primer for constructing pAK1B
pPSAEChlTP5	CATATAAATACCTTGAACATTTAA ATTTCAAAATGCTTGCTGCCCAGA ATAC	Binds to the 5' end of Chloroplast transit peptide of starch synthase with *pPSAE* overhangs	Gibson primer for constructing pAK9
pChlTPGFP3	TGTGTAGCTGGCAATGGCATCACG GCAGCAGAGACAGAAA	Binds to the 3' end of Chloroplast transit peptide of starch synthase with *5'GFP* overhangs	Gibson primer for constructing pAK9
pCHLGFP5	TTTCTGTCTCTGCTGCCGTGATGCC ATTGCCAGCTACACA	Binds to the 5' end of *GFP* with chloroplast transit peptide overhangs	Gibson primer for constructing pAK9
pGAPDHGFP3	CTTACTTTGACAGCGGCCGCTTAA GCCAACTCGTAGAAAG	Binds to the 3' end of *GFP* with *tGAPDH* overhangs	Gibson primer for constructing pAK9
pPSAEsecTP5	AATACCTTGAACATTTAAATTTCAA AATGTGGCACATATACTCCTC	Binds to the 5' end of periplasmic transit peptide of starch synthase with *pPSAE* overhangs	Gibson primer for constructing pAK10
SECtpGFP3	TGTGTAGCTGGCAATGGCATTTCCC TTGTTGCTTGGGCGC	Binds to the 3' end of periplasmic transit peptide of ars1 with *5'GFP* overhangs	Gibson primer for constructing pAK10d
SECtpGFP5	GCGCCCAAGCAACAAGGGAAATG CCATTGCCAGCTACACA	Binds to the 5' end of *GFP* with periplasmic transit peptide overhangs	Gibson primer for constructing pAK10

PcGFP5'	ATGCCATTGCCAGCTACACATG	Binds to 5' end of *GFP*	For verification of *GFP* presence
PcGFP3	TAAGCCAACTCGTAGAAAGCCT	Binds to 3' end of *GFP*	For verification of *GFP* presence
CNAT_Ver_5	ATGGCTTCTACAGGAACATTG	Binds to 5' end of clonNAT resistance marker	For verification of clonNAT resistance gene
CNAT_Ver_3	TTATGGGCATGGCATAGACAT	Binds to 3' end of clonNAT resistance marker	For verification of clonNAT resistance gene

Table S2: TG2 codon usage table*

Codon	Amino acid[1]	Per 1000[2]	Codon count	Codon	Amino acid[1]	Per 1000[2]	Codon count	Codon	Amino acid[1]	Per 1000[2]	Codon count	Codon	Amino acid[1]	Per 1000[2]	Codon count
UUU	Phe (F)	14.1	121	UCU	Ser (S)	28.7	181	UAU	Tyr (Y)	11.5	89	UGU	Cys (C)	4.5	42
UUC	Phe (F)	27.0	167	UCC	Ser (S)	14.5	93	UAC	Tyr (Y)	18.1	113	UGC	Cys (C)	11.1	74
UUA	Leu (L)	2.8	21	UCA	Ser (S)	7.3	67	UAA	Stop		10	UGA	Stop		5
UUG	Leu (L)	23.4	169	UCG	Ser (S)	5.1	50	UAG	Stop		2	UGG	Trp (W)	14.8	109
CUU	Leu (L)	15.4	118	CCU	Pro (P)	7.5	74	CAU	His (H)	8.6	84	CGU	Arg (R)	11.8	62
CUC	Leu (L)	17.1	106	CCC	Pro (P)	2.6	29	CAC	His (H)	9.4	73	CGC	Arg (R)	11.1	54
CUA	Leu (L)	1.7	13	CCA	Pro (P)	34.8	196	CAA	Gln (Q)	6.7	67	CGA	Arg (R)	4.6	40
CUG	Leu (L)	13.6	107	CCG	Pro (P)	3.0	33	CAG	Gln (Q)	27.3	161	CGG	Arg (R)	3.4	24

AUU	Ile (I)	26.7	203	ACU	Thr (T)	18.6	111	AAU	Asn (N)	15.7	133	AGU	Ser (S)	6.0	58
AUC	Ile (I)	22.6	161	ACC	Thr (T)	19.6	123	AAC	Asn (N)	27.5	160	AGC	Ser (S)	6.7	57
AUA	Ile (I)	4.5	41	ACA	Thr (T)	12.8	123	AAA	Lys (K)	13.7	116	AGA	Arg (R)	15.7	105
AUG	Met (M)	29.1	189	ACG	Thr (T)	5.5	53	AAG	Lys (K)	48.1	279	AGG	Arg (R)	6.7	65
GUU	Val (V)	25.3	172	GCU	Ala (A)	36.1	211	GAU	Asp (D)	28.0	224	GGU	Gly (G)	18.2	153
GUC	Val (V)	23.5	151	GCC	Ala (A)	28.2	172	GAC	Asp (D)	20.5	175	GGC	Gly (G)	8.6	89
GUA	Val (V)	5.2	48	GCA	Ala (A)	19.1	163	GAA	Glu (E)	15.2	144	GGA	Gly (G)	42.4	286
GUG	Val (V)	29.9	209	GCG	Ala (A)	6.1	49	GAG	Glu (E)	39.4	255	GGG	Gly (G)	9.4	92

*Calculated using 17 predicted proteins by using CAI calculator [42]

[1] The letter in parenthesis represents the one-letter code for amino acids

[2] represents the average frequency (from 17 proteins) this codon is used per 1000 codons
